# Design of Virtual Reality Exergames for Upper Limb Stroke Rehabilitation Following Iterative Design Methods: Usability Study

**DOI:** 10.2196/48900

**Published:** 2024-01-11

**Authors:** Julian Felipe Villada Castillo, Maria Fernanda Montoya Vega, John Edison Muñoz Cardona, David Lopez, Leonardo Quiñones, Oscar Alberto Henao Gallo, Jose Fernando Lopez

**Affiliations:** 1 Faculty of Basic Sciences Department of Physics Universidad Tecnologica de Pereira Pereira Colombia; 2 Department of Human-Centered Computing Exertion Games Lab Monash University Melbourne Australia; 3 Department of Systems Design Engineering University of Waterloo Waterloo, ON Canada; 4 Engineering Faculty Universidad Tecnologica de Pereira Pereira Colombia; 5 Medicine Faculty Universidad Tecnologica de Pereira Pereira Colombia

**Keywords:** stroke, user-centered design, exergame, design, virtual reality, playtest, upper limb rehabilitation

## Abstract

**Background:**

Since the early 2000s, there has been a growing interest in using exercise video games (exergames) and virtual reality (VR)–based interventions as innovative methods to enhance physical rehabilitation for individuals with multiple disabilities. Over the past decade, researchers and exercise professionals have focused on developing specialized immersive exercise video games for various populations, including those who have experienced a stroke, revealing tangible benefits for upper limb rehabilitation. However, it is necessary to develop highly engaging, personalized games that can facilitate the creation of experiences aligned with the preferences, motivations, and challenges communicated by people who have had an episode of stroke.

**Objective:**

This study seeks to explore the customization potential of an exergame for individuals who have undergone a stroke, concurrently evaluating its usability as a technological tool in the realm of physical therapy and rehabilitation.

**Methods:**

We introduce a playtest methodology to enhance the design of a VR exergame developed using a user-centered approach for upper limb rehabilitation in stroke survivors. Over 4 playtesting sessions, stroke survivors interacted with initial game versions using VR headsets, providing essential feedback for refining game content and mechanics. Additionally, a pilot study involving 10 stroke survivors collected data through VR-related questionnaires to assess game design aspects such as mechanics, assistance, experience, motion sickness, and immersion.

**Results:**

The playtest methodology was beneficial for improving the exergame to align with user needs, consistently incorporating their perspectives and achieving noteworthy results. The pilot study revealed that users had a positive response. In the first scenario, a carpenter presents a game based on the flexion-extension movement of the elbow; the second scenario includes a *tejo* game (a traditional Colombian throwing game) designed around game mechanics related to the flexion-extension movement of the shoulder; and in the third scenario, a farmer challenges the player to perform a movement combining elbow flexion and extension with internal and external rotation of the shoulder. These findings suggest the potential of the studied exergame as a tool for the upper limb rehabilitation of individuals who have experienced a stroke.

**Conclusions:**

The inclusion of exergames in rehabilitation for stroke-induced hemiparesis has significantly benefited the recovery process by focusing on essential shoulder and elbow movements. These interactive games play a crucial role in helping users regain mobility and restore practical use of affected limbs. They also serve as valuable data sources for researchers, improving the system’s responsiveness. This iterative approach enhances game design and markedly boosts user satisfaction, suggesting exergames have promising potential as adjunctive elements in traditional therapeutic approaches.

## Introduction

### Background

In recent years, technological advances have influenced motor rehabilitation interventions for survivors of stroke, with the introduction of exergames, known as “serious games for health,” which help motivate individuals in their rehabilitation [1-4]. However, the development of such exergames needs to consider users’ needs and rehabilitation goals [5].

Virtual reality (VR) immersive [6] systems have become increasingly popular in rehabilitation, as they offer immersive and engaging activities, improving motivation and skill acquisition [7]. Nevertheless, systematic reviews have noted that most VR apps primarily focus on balance and gait, with limited attention to upper extremity rehabilitation [8,9].

Efforts have been made to design exergames tailored for survivors of stroke, but challenges remain, including limited user involvement and lack of immersive VR integration [10,11].

This study aims to address these challenges by designing a VR-based upper limb rehabilitation exergame using a user-centered approach, involving survivors of stroke in the design process and conducting playtests with an immersive VR setup [12]. The methodology aims to improve interdisciplinary collaboration and facilitate the involvement of clinicians in the design process [13,14]. The primary objectives are to provide personalized upper arm physiotherapy for survivors of stroke through an improved VR exergame and to assess its usability through user feedback [15]. This work encourages collaboration among clinicians, researchers, and designers to create an engaging rehabilitation exercise that complements the recovery process for survivors of stroke, ultimately enhancing their quality of life.

### Related Work

#### VR-Based Physical Rehabilitation for Stroke

Experts in rehabilitation, kinesiology, and neuroscience are integrating VR systems with exergames to enhance the appeal and effectiveness of rehabilitation processes [16]. Early studies, such as those by Henrique et al [17] and Burke et al [18], demonstrated the positive impact of exergames on balance, gait, and upper limb motor function in patients with stroke, highlighting improved therapy adherence [14,16-20]. However, systematic reviews have indicated that most VR apps for after-stroke therapy primarily focus on balance and gait, with limited attention to upper extremity rehabilitation [3,21,22]. To address this gap, we aim to evaluate the potential of an exergame for upper limb rehabilitation using immersive VR systems [22].

In addition, prior research has shown that complementing or replacing standard rehabilitation with VR-based rehabilitation can result in significant improvements in gait speed, balance, and mobility in patients with stroke [3,17,21-23]. Our work aims to contribute to the development of guidelines for using VR-based rehabilitation in conjunction with conventional therapy, with a focus on upper limb rehabilitation.

Although some researchers, such as Reis et al [10], Leung et al [11], and Horsham et al [24], have proposed methodologies for developing specific exergames for stroke rehabilitation, there is still limited knowledge regarding immersive VR-based designs targeting upper limb rehabilitation [10,11,24]. Therefore, we intend to involve survivors of stroke in an iterative playtesting process to develop an upper limb VR-based rehabilitation system and bridge this gap.

#### Playtesting as an Iterative Design for Stroke

This section covers research related to the use of playtesting as an iterative user-centered design (UCD) methodology. UCD has played a significant role in the development of games for rehabilitation and overall health [25,26], as it is a methodology that allows active participation of the target population in the system’s prototyping process. UCD, applied in game design, often advocates for an interactive and participative methodology that includes multiple playtests with end players. Playtesting is an activity carried out with potential users or players who interact with game prototypes developed in the early stages, making it easier to gather individual opinions and ideas that contribute to improving the gameplay aspects of exergames during their development [27,28]. Playtesting is a key and standardized methodology used in game studios to iterate and systematically improve games before they are released to the public [29].

A relevant example is the work of Duval et al [30], who conducted a collaborative study with 14 clinicians, focusing on therapeutically validating the game based on their opinions rather than those of users. Duval et al [30] obtained significant findings by addressing the adoption of therapy and personalizing it according to the characteristics valued by medical professionals. In contrast, other UCD works, such as the study by Aguilar et al [31], have not used playtesting but have used usability tests involving scales and flow state questionnaires. Findings from 3 years of experience with exergames developed for older adults using UCD methods concluded that devoting the key to engaging with end users and considering feedback and opinions can be considered the best practice guide for the development of therapeutic games [32]. We believe that playtesting can be beneficial for the design process of games for health, as it is strongly recommended to involve the target audience during the game design and development processes. By doing so, developers increase the likelihood of creating games that consider the specific preferences, motives, and characteristics of survivors of stroke in need of physical therapy [21]. By including survivors of stroke in interactive playtesting and, consequently, in enhancing a VR exergame, we begin to understand how this design methodology affects the subsequent use of the exergame as a therapeutic tool.

## Methods

In this section, we introduce the interdisciplinary team that worked on the improvement of the VR exergame we used in the playtesting session and the pilot study, as well as the description of the VR exergame. Furthermore, we present the playtesting methodology and the pilot study methodology.

### Interdisciplinary Team

The structured design team was composed of an expert clinical physiatrist who advised the movements that users with stroke are likely to perform from a clinical viewpoint; a physiotherapist who provided permanent follow-up in all sessions with the users; a designer of exergames who helped implement the UCD methodology to have clear game mechanics; an expert in biomechanics who analyzed ranges of movement, postures, and gestures; a user experience researcher who organized all sessions with the users; 2 professional game programmers who created the game prototypes; and 2 users who experienced stroke episodes and interacted with the system and based on the answers they gave us an improved exergame. For 2 months, this group convened weekly to discuss the exergames’ requirements, technologies, and overall scope of the project. The discussions were centered on defining the activities in the internet-based environment and strategies for the recruitment of potential users. At the end of the design process, the group of game developers with programming experience used the Unity game engine (Unity Technologies) to materialize the ideas.

The main topics addressed by the interdisciplinary team were (1) the definition of the main objectives and roles of the project; for example, project management was assumed by the exergames designer, and the user experience researcher assumed the role of project manager and conducted most of the fieldwork; (2) socialization of playtesting activities, including user recruitment and experimental protocol; and (3) reconsideration and further adjustment of game design elements, such as game mechanics and their mapping with therapeutic objectives.

### Design of the VR Exergame

#### Prior Design of the VR Exergame

We performed a rapid contextual design based on a previous study, where user profiles were defined using user personas [33]. In this study, we characterized 4 persona roles that distinguish them as gamers: apathetic, empathetic, beginner, and experienced. Specifically, we used the results of the user modeling process to define certain game elements. For example, we found that users showed interest in sports games. Hence, body interaction familiar with certain sports games was an important requirement to be integrated into the exergames. In addition, users were comfortable with game content related to their daily lives. Therefore, incorporating cultural activities into the exergames could be a promising approach. In addition, in this prior study, we considered the following clinical requirements when developing the VR exergame [34,35].

Population specificity: according to previous studies [36], most users who have experienced a stroke are older than 40 years, and few are younger than 30. These studies also showed that the older population has little experience with VR. In contrast, therapist experience suggested that ranges of motion vary across users who have experienced a stroke. Therefore, designers should be careful when adapting internet-based therapy to a wide range of capabilities [37].

Motor learning: different principles of motor recovery should be considered in the creation of the activities to be performed within the exergames, such as a meaningful task, intensive and repetitive practice, movements close to the normal range, muscle activation that drives the practice of movement, and variability and progression of training [10,38,39].

Rehabilitation movements: the movements suggested by clinicians to perform upper limb physical therapy in paretic users are listed in Table 1. These are shoulder flexion and extension, elbow flexion and extension, and Kabat diagonals. Kabat diagonals are internal and external rotation movements of the shoulder. According to Della Tommasina et al [40], a repetitive process of these movements is necessary to perform physiotherapy, from which a more effective range of motion recovery will be obtained [41]. The elbow and shoulder are the upper limbs’ main joints, articulating the arm’s largest segments. These joints require a greater range of motion in flexion and extension and often affect and limit arm motion when a stroke episode occurs. Hence, we decided that users in a seated position should perform different arm movements while playing the VR-based rehabilitation exergame, targeting multiple possible physical rehabilitation needs in the upper limbs of people with stroke.

**Table 1:** Rehabilitation movements proposed for the exergame.

	Movement	Application	Action in the exergame
Elbow	Flexion and extension	Improvement in the width of movement in daily life activities	Destroying blocks with a hammer
Shoulder	Flexion and extension	Improvement in the width of movement in daily life activities	Throwing *tejos*
Elbow	Interior and exterior extension	Improved range of motion in the daily life activities	Cutting grass with a machete

Complementary therapy: we propose a therapy that uses the VR-based rehabilitation exergame to complement the rehabilitation process instead of replacing the traditional one, such as the one proposed by Goncalves et al [42]. We are confident that users who have experienced a stroke will play an exergame with engaging activities because they are developed based on their needs and motivations. Therefore, the exergames will allow a disruptive experience different from conventional therapies, generating interest, excitement, and willingness to carry out their rehabilitation process without neglecting the conventional therapy recommended by clinical specialists [38]. Considering the rapid contextual design and the clinical requirements named in this study, in a prior study, we developed a VR exergame following the well-known game design methodologies by Schell [29], as we contextualize in the following subsection.

#### Motion Health VR: VR Exergame for Stroke Rehabilitation

In a prior study, we established a game design concept and game mechanics using the methodology proposed by Schell [29] and a complete contextual design conducted with users with stroke. This study covers a more systematic and complete description of the playtesting sessions conducted with players with stroke to iterate and improve the game based on the initial concept. Knowing the preferences, ages, and profiles of potential users, we decided to explore a design concept for the cultural regions of Colombia (the Caribbean, Pacific, Andean, Orinoco, and Amazon). We discovered that older adults are inclined to engage in activities in the countryside and typical and authentic Colombian games. We discussed this exergame concept with the clinicians of our design team, gathering feedback regarding the potential movements to be performed, particularly in the context of mapping them for stroke rehabilitation therapies. Considering the scope of the project and previous research conducted with local users, development capabilities, and timelines, we decided to start by developing a game design concept related to the activities of the Colombian Andean region. The Andean region is the central region of Colombia and has crosscutting activities that are representative of the entire country and run throughout the central Andes. The population of this region practices sports, such as *sapo* and *tejo* (throwing games), rowing, and other more well-known sports, such as basketball and boxing. They also engage in other daily activities, such as fruit picking, horseback riding, and bush cutting [43]. Aligned with these cultural activities, we designed game scenarios that focused on a local setting using Colombian games. The design team analyzed existing VR games to establish game mechanics that could involve desired rehabilitation movements, always considering the player’s motivators and needs. This analysis facilitated communication between the team of clinicians and specialists and the design and development team while also helping to specify the activities that would be familiar and engaging to the users. Therefore, we called the VR exergame “Motion Health VR.” The exergame comprises 3 main scenes in which players must develop 3 different activities: hammering, throwing a metal disk (a traditional Colombian game called *tejo*), and cutting bushes while riding a horse.

**Figure 1:**
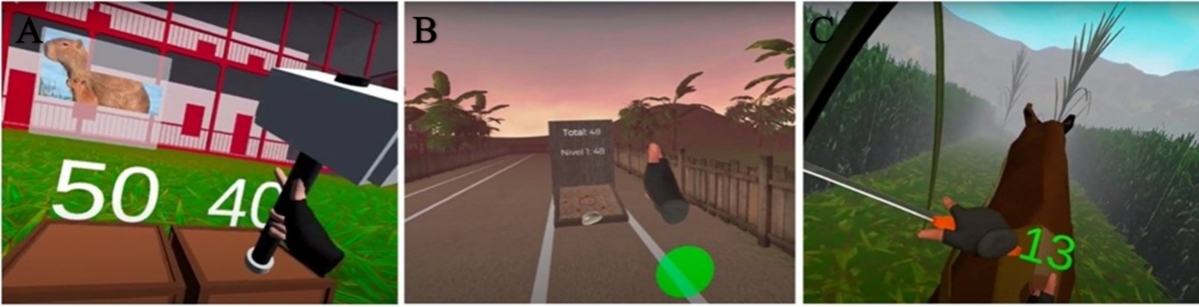
The presented scenarios of the Motion Health VR exergame with (A) a carpenter, (B) a throwing activity, and (C) a farmer, based on the movement of the Kabat diagonals.

The exergame presents 3 meticulously crafted scenarios, each aligned with its unique reference to Figure 1. In the “carpenter” scenario (Figure 1A), players engage in a dynamic elbow flexion and extension challenge, wielding a hammer to systematically crush boxes that vary in color and size, demanding specific ranges of motion. Players must skillfully adjust their proximity to the boxes to adapt to this diverse challenge, seamlessly weaving in back-and-forth and crossbody-reaching motions. Between the box-smashing activity, a captivating puzzle gradually unveils itself, featuring distinct Andean wildlife. Upon completing the activity, players earn the gratifying experience of visualizing the completed animal puzzle. As players enhance their hammering skills, the game dynamically escalates in difficulty either by increasing hammering frequency or by reducing box sizes. In the second scenario, inspired by Colombia’s traditional *tejo* game (Figure 1B), players embark on a shoulder-focused flexion and extension adventure, mirroring the popular sport played nationwide. Throwing a metal disk toward an explosive target known as a *mecha* on a clay court, players must adjust their shoulder movements according to the target’s distance, finetuning their range of motion for precise throws and aiming to maximize target hits with minimal repetitions. In the third scenario, the “farmer” (Figure 1C), players are challenged with a multifaceted movement that combines elbow flexion, extension, and internal and external rotation of the shoulder, akin to Kabat diagonals. In this rural setting, players ride an internet-based horse while wielding a machete, a staple tool in the Colombian countryside, tasked with clearing the obstructive bushes that appear on both sides of the road. With one arm gripping the machete and the other resting on the horse’s rein, players face escalating challenges as the game progresses.

#### Playtesting Sessions

Iterative game design involves playtesting sessions with end users, who will shape the game features before its final deployment. The main objective of playtesting sessions is to iterate different playable prototypes to improve the overall playability of the game, thus increasing the likelihood of adoption. In addition, playtesting allows the researcher to assess the ability of potential players to perform the proposed activities and understand game feedback. We developed playtesting sessions using the VR exergame designed in a prior work.

The playtest sessions were guided by a researcher accompanied by a clinical specialist who helped contact different users who had experienced a stroke. Owing to the COVID-19 pandemic, visits to each user were scheduled in such a way that biosafety protocols were maintained (eg, distancing, constant use of masks, and hand and footwear disinfection). Upon arrival at the agreed location, stakeholders performed the recommended distancing protocols. Then, the researcher prepared the experimental protocol, which consisted of setting up a table, a chair without a hand rest, and verifying the internet connection. Prototypes of the exergames were developed before the playtesting sessions and ported to the VR headsets (Oculus Rift in the first 4 iterations and Oculus Quest in the final version). Disposable headset protectors and cleaners were used to maintain biosafety measures. Table 2 presents the structure of the playtest sessions.

**Table 2:** Protocol for conducting playtests.

Actions	Considerations	Time (min)
Brief introduction of the dynamics of the session and interaction between the researcher and the user with stroke to obtain informed consent	—^a^	10
A quick explanation of how the VR^b^ system works and what to expect from the activity	Preparation and arrangement	5
System implementation (HMD^c^, headphones, and controls)	—	—
Free play or natural interaction with the system	Manifestation of difficulties, in real time if necessary	5
Receive feedback or explore ideas while users play	Formulate the questions established for the session	10
Conclude the session with questions about the experience	Questions	10

^a^Not available.

^b^VR: virtual reality.

^c^HMD: head-mounted display.

A total of 4 playtesting sessions involving 9 end users who had experienced a stroke were conducted. Each session was performed with a minimum of 2 users chosen considering their availability (Multimedia Appendix 1 lists the users who participated in each session and their demographic information). After playtesting, we recorded a video summarizing the session and documented a brief analysis that was subsequently discussed with the research and design teams. After each playtest, the team held a general meeting where all the discussions were presented. The subsequent playtest was scheduled after the implementation of the suggested game changes.

### Pilot Study: Evaluation of the Game Experience and Usability of the Exergames

After conducting game playtests and completing a playable prototype of the VR exergame (4 iterations), we decided to carry out a pilot study to evaluate the usability of the game with a group of users who had experienced a stroke, in which 2 users who participated in one of the 4 iterations were part of the pilot study group.

We conducted a 20-minute session that was part of the rehabilitation therapy in which users who had experienced a stroke played the iterated version of the Motion Health VR exergame. We ported the final version of the game to the standalone VR headset, Oculus Quest 2, as it has several advantages, such as being wireless, comfortable, and having a high image resolution.

### Users

This usability study was developed with 10 users who had experienced a stroke contacted through the clinician and therapist of the design team. We chose this sample size conveniently, considering the availability of users, which was very limited. In contrast, the small sample size allowed us to follow the biosafety protocols required for the COVID-19 pandemic, which was still ongoing in Colombia at the time of the study. The inclusion criteria for the study were being aged >50 years, having experienced a stroke and having hemiparesis or monoparesis, being able to read and write, not having serious vision problems (eg, strabismus), and not having diagnosed cognitive disabilities (eg, dementia).

### Ethical Considerations

The bioethics committee of the local university approved this study, which was also approved by the bioethics committee of a local rehabilitation center (52–050623). Users volunteered for this study and agreed to participate by signing an informed consent form.

### Usability Study

Two questionnaires (instruments) were used to assess the game user experience immediately after interacting with the immersive game.

#### Virtual Reality Neuroscience Questionnaire

The Virtual Reality Neuroscientific Questionnaire (VRNQ) measures the quality of user experience, game mechanics, and in-game assistance. It comprises 20 questions, each scored on a Likert-type scale ranging from 0 to 5 [44]. The advantage of using this questionnaire is that it provides the limits to assess the suitability of the software in VR [44]. VRNQ produces a total score that reflects the overall quality of the VR software and 4 categories as follows: (1) game experience, where the level of immersion and pleasure of the experience are evaluated; (2) game mechanics, where user interaction in the internet-based environment is evaluated; (3) game assistance, where the exergame instructions, indications, arrows, and labels are evaluated; and (4) motion sickness, which evaluates whether you experience nausea, disorientation, fatigue, and instability.

#### Immersive Tendencies Questionnaire

The Immersive Tendencies Questionnaire (ITQ) determines the differences in an individual’s tendencies to experience immersion and presence after interacting with a VR scenario. ITQ comprises 18 questions rated on a Likert scale from 1 to 7, resulting in a possible score ranging from 18 to 126. In the original study, the mean score of the samples was 76.66 [45]. This questionnaire is considered a standard in VR research and has been widely used in different applications [22]. A user with a positive immersive tendency based on the ITQ score is likely to experience higher levels of VR presence, which has been associated with better task performance [22,41,46]. This questionnaire is useful because it allows the evaluation of immersion in a way that does not depend on the specific internet-based environment, making it possible to determine independently if an internet-based environment performs poorly or if the statistical sample has low immersion trends. Some ITQ questions are as follows:

- Does it often happen that while daydreaming, you forget what is happening around you?
- Does it happen to you that you are so engrossed in a movie that you forget what is happening around you?
- Do you identify with television characters?
- When you use an exergame, does it occur to you that you feel like you are inside the game instead of sitting down using the controller?
- Do you stay scared for a while after watching a scary movie?

### Experimental Setup

The researcher and the physical therapist held the interaction session at each participant’s home, where they chose a comfortable space to set up the VR system. The setup consisted of a chair in which the user with stroke was seated with the Oculus Quest 2 wearable headset and its respective wireless controllers. The researchers were able to see what the players were doing via the official Meta Quest app using an electronic tablet in real time.

### Protocol

The users began the pilot study session seated, using the headset and holding the controllers. We conducted the session in the following order. The user who had experienced a stroke performed an upper body warm up for 5 minutes, guided by the physical therapist. The researcher and physical therapist prepared the user for the game by helping them put on the VR system. The researcher started the exergame, which was presented throughout the session, accompanied by a physiotherapist. After the interaction, the user who had experienced a stroke completed the 2 proposed questionnaires. Given the biosafety regulations established by the Colombian government because of the COVID-19 pandemic, all those involved in the sessions always wore masks. In addition, the researcher cleaned all VR and mounting elements each time they were used.

### Data Analysis

The questionnaires were scored following the instructions of previous works. Descriptive statistics, such as mean and SD, were calculated and reported [47,48].

## Results

### Overview

The results are presented in 2 subsections. The first subsection details the transformation process of the game after conducting 3 playtesting sessions and iterations involving the end users and the interdisciplinary design team. The second subsection presents the preliminary results of a pilot study evaluating the user experience of the final game with a group of 10 players with stroke.

### Playtesting

The following subsections detail the results of the iterative design process of the Motion Health VR exergame, reporting the details of the playtesting sessions and the modifications made to each iteration. The overall objectives of the playtest were to validate the acceptance and playability of users with stroke and to explore whether they were able to perform the activities proposed in the VR scenarios and game mechanics. In addition, analysis of errors and optimization of game mechanics were crucial to improve playability. A total of 9 users with stroke were involved throughout the 4 playtesting sessions conducted (Multimedia Appendix 2), focusing on certain game elements (eg, mechanics and esthetics) via playable prototypes and reporting back to the design team.

### Analysis of Playtest 1

This version of the exergame was created to test the first 2 scenarios. Players 1 and 2 (U1 and U2) participated in this session following the protocol in Table 2. We used a VR-ready laptop and an Oculus Rift headset with controllers. The playtesting goals were to evaluate the appropriateness of the proposed range of movement for hammering and throwing the disk and to explore button combinations for performing the activity using the controllers. We found that (1) the game should consider different scales of spasticity to provide a more adaptive experience [49]; (2) the buttons should be suspended from their functions to avoid triggering involuntary functions; and (3) a rest period should be granted to the user because, as recommended by the physiotherapist, long periods of exercise generate symptoms of fatigue.

### Analysis of Playtest 2

**Figure 2:**
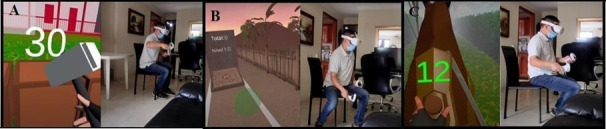
The final version of each scenario of the Motion Health VR exergame: (A), the carpenter scenario, (B) the tejo scenario, and (C) the farmer scenario.

The objective of these playtests was to test the modifications introduced in the first 2 scenarios based on the considerations in the Analysis of Playtest 1 subsection. Players U3, U4, and U5 participated in this playtest following the protocol in Table 2. A VR-ready laptop and an Oculus Rift headset with controllers were used. Figure 2 shows the evolution of the 2 scenarios after the first playtest, showing the improvement in content according to the real scenarios where these activities were performed.

Users with stroke reported an improvement in the simplicity of the interaction, as they found it much easier to perform the movement owing to an initial calibration of the position added to the 2 scenarios, which adjusts the player’s position concerning the internet-based surroundings, ensuring objects are at a reachable distance. In addition, we found that (1) the objects in each scenario should be in a static position; thus, people can avoid unnecessary displacements within the internet-based space that can generate dizziness; (2) we need to improve the auditory feedback of scenarios to create an immersive experience; and (3) we need to improve the calibration scene to allow players with low mobility to perform the tasks.

### Analysis of Playtest 3

The third scenario was prototyped, and the game mechanics were ready for playtesting. The objective of this playtest was to test whether players could easily understand and interact in the farmer scenario by performing the proposed movements, that is, riding the horse while holding the rein with the unaffected arm and cutting bunches with the machete using the affected arm. Players U6 and U7 (Multimedia Appendix 3) participated in this test following the protocol in Table 2. We used a VR-ready laptop and an Oculus Rift headset with controllers. We found that (1) it would be useful to place the avatar on the horse from the beginning and (2) the game should allow cutting bushes to be performed using both arms and provide adequate time to switch the game controller between hands because the players become tired after certain repetitions.

### Analysis of Playtest 4

After iterating each scenario and exploring potential pitfalls and interaction errors, a final playtesting session was scheduled to test the overall functioning of the integrated system. This prototype of the exergame presented an embellishment of the contents (Figure 2), which was an improvement in the overall esthetics of the game. In addition, a structured exercise session was recommended by the clinical rehabilitation experts, following a 15-minute session (similar to other studies of the same nature [50]). Therefore, the 3 scenarios were presented in sequence and switched after 5 minutes (approximately). Players U3, U4, and U5 participated in this test following the protocol in Table 2. We used a VR-ready laptop and an Oculus Rift headset with controllers.

We found that (1) scene transitions should be smoothed and (2) the game should implement rest periods between each scenario, as users still manifested mild fatigue from performing so many repetitions while preparing for the next mechanic.

Finally, we integrated the above recommendations into the scenarios and developed the final prototype of the Motion Health VR exergame.

### Evaluation of Game User Experience

This section presents the results of evaluating the game user experience of the co-designed Motion Health VR exergame involving 10 users with stroke (Multimedia Appendix 4). For this part, the game was modified to a more portable, standalone, and easy-to-use headset, the Oculus Quest 2. Only the final deployment platform was changed (from wired to wireless VR), and no other changes were made. The questionnaires were administered at the end of the session, asking users to rate their experience in a wide range of aspects following the ITQ and VRNQ.

### Virtual Reality Neuroscientific Questionnaire

The results of administering VRNQ are reported as average values with SD (Table 3). Each category had a maximum of 35 points. Gaming experience was rated at a mean of 24.8 (SD 4.5), game mechanics mean 23.8 (SD 5.5), game assistance mean 23.9 (SD 5.5), and motion sickness (inversely proportional), mean 31.1 (SD 5.6). Consequently, the maximum possible general score for this test was 140, in which the exergame Motion Health VR obtained a mean of 103.6 (SD 19.4). This level of quality is considered more than adequate as it exceeded 100 points. From this, it can be concluded that the users experienced a high level of immersion during their video game experience, and the quality of the Motion Health VR exergame obtained a general average of mean 103.6 (SD 19.4), which is considered an adequate quality because it exceeded 100 points [47]. On the basis of this result, we observed that users had a high immersion index; the experience with the exergames was very pleasant; and the quality of the graphics, sound, and technology, in general, was perceived as very positive. Finally, the system showed the best results in the motion sickness index, which shows that exergames did not cause major side effects associated with cybersickness or nausea [47,51].

**Table 3:** Virtual Reality Neuroscientific Questionnaire (VRNQ) categories.

VRNQ categories	Score, mean (SD)
Game experience	24.8 (4.5)
Game mechanics	23.8 (5.5)
Game attendance	23.9 (5.5)
Motion sickness	31.1 (5.6)

### Immersive Tendencies Questionnaire

ITQ was used to evaluate users’ immersion experience and presence following their engagement with the exercise. The overall ITQ score averaged 60.8 (SD 11.6), signifying that participants with a history of stroke perceived relatively low to moderate levels of immersion and enjoyment (Table 4). In terms of concentration (mean 22.3, SD 2.9, with a maximum score of 35), users consistently achieved high scores, indicating that the game effectively captured their attention.

Regarding immersion (mean 17.9, SD 4.7, with a maximum score of 35), users reported a sense of engagement with the game. In terms of emotions (mean 14.6, SD 5.1, with a maximum score of 28), the findings suggest that users developed a strong emotional connection with the game [33,51,52].

**Table 4:** Immersive Tendencies Questionnaire (ITQ) categories.

ITQ categories	Score, mean (SD)
Concentration	22.3 (2.9)
Immersion	17.9 (4.7)
Emotion	14.6 (5.1)
Enjoyment	6.1 (2.2)

## Discussion

### Principal Findings

This study summarizes our efforts to report methodological approaches extensively used in game design that have a significant value when used to design VR exergames for stroke. We also showed the results of a preliminary usability test conducted involving 10 users with stroke who played the Motion Health VR exergame after completing 4 iterations using playtesting sessions. Overall, the VR exergame exhibited medium-to-high levels of game user experience and low levels of perceived symptoms associated with VR, such as nausea and dizziness. Moreover, regarding enjoyment, users expressed a high willingness to participate in therapies and continue with the sessions. The user experience questionnaires showed that users experienced increased immersion, emotional connection, and enjoyment with the VR exergame, although the concentration remained consistent. These results are consistent with previous research using immersive VR in older adults [53] and people with stroke [54,55]. Furthermore, we have carefully reported the methodological aspects related to playtesting sessions with users with stroke and specific procedures to conduct such sessions. From the playtesting sessions, we can extract the value of evaluating prototypes in the early stages of the game design process because this prevents researchers from struggling with complex interactivity and usability issues later during the trials. As reported in the study by Toro et al [56], early involvement of end users in VR systems for exercise promotion is a desired practice, and it is not commonly used among those creating custom-made exergames for older adults [57-59].

### Playtesting as a Tool for Iterative Design

Playtesting is a part of the iterative design methodology used in different UCD approaches [51,60]. In our case, we performed playtest with several users who had experienced a stroke, which allowed us to improve the content, playability index, and game mechanics from an ergonomic approach to provide greater user comfort. Although prior designs of physical rehabilitation games for stroke have involved UCD [61], involving users with stroke in playtesting and follow-up sessions is not common [50]. We suggest that designers consider including playtesting with users with stroke because, in terms of rehabilitation therapy performance, playtesting revealed important details, such as the importance of performing a calibration stage or removing the buttons and other interactions with the VR equipment. We found that this stage provides exergames with the characteristics to adapt to the physical needs of each user, such as the range of motion and spasticity scale of each user [62]. In the context of serious games, the importance of adaptive games has increased, as every user has different requirements [62].

Furthermore, the playtesting methodology allowed us to strengthen relationships with all stakeholders, from developers to clinicians. This aligns with the findings of previous studies that have emphasized how involving multiple stakeholders in the design process leads to a more suitable and user-centered prototype [63]. The importance of maintaining close relationships with stakeholders has also been underscored, and this study reaffirms the relevance of this challenge in the successful implementation of playtesting. Effective collaboration between developers and clinicians, driven by the willingness and availability of both parties, has been a recurring theme in the literature [62]. Previous studies have pointed out the pivotal role of this relationship in the success of VR rehabilitation programs following stroke.

In particular, we noticed that playtesting allowed a stronger relationship with patients with stroke, increasing their willingness to participate in future studies. Although other researchers have reported difficulty finding specific populations to be involved in studies [64,65], considering our final results, we highly recommend that research teams plan to conduct multiple playtests before conducting studies.

Finally, we consider that after performing 4 playtests, based on the observations of the users who had experienced a stroke and the recommendations of the clinicians, an optimal version of the exergame was obtained, which could also be used by designers to facilitate piloting and prevent errors during data collection. As mentioned in this study, the inclusion of playtests and the collaboration of specialized professionals in programming and design align with the best practices recommended by previous research [66].

### Usability of UCD VR Games in Patients With Stroke

We observed that ITQ and VRNQ scores were below the expected mean, as reported in the Results section. Similar results have been reported previously because there were some concerns about the usability of VR in older adults, including those who have had strokes. A systematic review of clinical and research applications of VR in older people identified usability issues, such as discomfort, cybersickness, and difficulty with the equipment [67]. Therefore, based on the current results, the design team must improve the gameplay mechanics and usability of the VR exergame to achieve better results in gameplay experience metrics before the subsequent trial. Nevertheless, the system scored high in usability, as its overall score was higher than expected (100 points). Furthermore, as our results showed that the VRNQ category with the highest score was motion sickness, meaning that users who experienced a stroke felt little nausea, our work aligns with studies using similar VR apps [8,20,47]. These usability results can guide other exergame designers to adjust their apps to suit older adults.

### Use of Interactive Technology for Telehealth Care

The use of portable and autonomous technologies, such as the Oculus Quest 2 headset, during the pandemic has been an innovative response supported by this research [68]. This reinforces the notion that virtualization of health care, driven by technology, is becoming increasingly important. A recent review describing the promising landscape of telerehabilitation tools aided by serious games for upper limb stroke rehabilitation highlights the evidence of efficacy, the need for further research in this area, and the promise of digitally connected games to complement conventional rehabilitation [69]. Nevertheless, although VR has never been more accessible before, the reality is that the cost-effectiveness of its use in telehealth programs in both rural and urban areas in Latin America is still very limited [70]. In summary, our study contributes to emerging efforts in which interdisciplinary collaboration and the use of innovative technology during times of crisis, such as the pandemic, continue to draw a research pathway in this continually evolving field [63,71,72].

### Limitations

We developed this study between August 2020 and June 2021, when most rehabilitation centers were closed owing to COVID-19 pandemic restrictions. Therefore, accessing users with stroke was a difficult task that we overcame with the help of the therapists. They provided us with the contact list of their former users, and we contacted them personally. Notably, although we took all safety precautions, people with stroke feared contagion and only a couple of them participated in the playtesting sessions. We acknowledge the lack of homogeneity in the sample of users who participated in the playtesting because stroke is a condition caused by several factors and affects both sexes, and users who have experienced a stroke tend to have a wide range of ages and ethnicities. The small sample size may limit the generalizability of our findings. Moreover, this limitation was difficult to address because of the COVID-19 pandemic restrictions that were under regulation when we developed this study. That is why, for the pilot study, we limited the users’ age to >50 years. VR is a technology that is constantly changing and improving, in the sense that we started playtesting with the Oculus Rift headset and then moved on to using the Oculus Quest 2 for the pilot study because of its portability advantages. We overcame these technological changes because of the cross-platform features offered by the game engine used (Unity).

However, VR content development is a challenge when designing deployable solutions. Nonetheless, despite the pandemic situations in which this study was developed, the iterative design and preliminary study were carried out owing to the implementation of portable and autonomous tools, such as the Oculus Quest 2 VR headset, which are becoming increasingly important in the virtualization of health care delivery.

The duration of the usability study was very short, and users only interacted with the final game in a single session lasting approximately 15 minutes. A short-term study may not adequately capture the long-term benefits or challenges of using VR exergames for stroke rehabilitation. We plan to extend this initial pilot study and conduct a single-arm longitudinal study involving a similar group of users for 12 sessions for 3 months. Furthermore, this study did not include a control group for comparison. Without a control group undergoing traditional rehabilitation methods, it is challenging to conclusively attribute improvements to the VR exergame alone. Finally, although the study emphasizes the iterative design process and user feedback, it is difficult to know how these design changes directly affect the rehabilitation outcomes. Future studies with this game should include rehabilitation outcomes such as upper limb range of motion and spasticity levels.

### Conclusions

Our research has conclusively demonstrated that creating VR exergames for stroke rehabilitation by involving end users early in the design stages brings advantages such as reducing interaction errors and unnecessary game design elements that do not contribute to the therapy. UCD is highly recommended as a design methodology for creating games specifically tailored to the rehabilitation of users who have experienced strokes. The results of this study support the effectiveness of multiple playtesting in producing therapeutic games that align with the needs and abilities of users with stroke, such as creating familiar internet-based environments and activities and removing unnecessary motion that could lead to motion sickness. This conclusion underscores the importance of adopting a patient-centered approach in the development of medical apps and technologies for rehabilitation. Our findings have yielded promising results regarding the use of immersive VR in the context of upper limb stroke rehabilitation. Users who immersed themselves in internet-based environments using custom-built exergames showed good levels of immersion and enjoyment and reduced levels of perceived nausea or dizziness. These results suggest that VR technology holds potential as a therapeutic tool for the treatment of users with stroke-related impairments, especially for at-home therapies. However, further research and long-term follow-up are required to fully understand the scope and limitations of this technology in the rehabilitation of this user group. The use of playtesting as an iterative tool for enhancing video game design enables comprehensive interaction with the user. This interaction allows for genuine customization of the therapy, leading to the development of a video game tailored specifically for users who have experienced stroke.

## Appendix

Multimedia Appendix 1 Users in the playtests.

Multimedia Appendix 2 Demographic data of the user’s study pilot.

Multimedia Appendix 3 Changes made throughout the iteration process of the playtests.

Multimedia Appendix 4 Scenario sketches.

## Abbreviations

ITQ: Immersive Tendencies Questionnaire
UCD: user-centered design
VR: virtual reality
VRNQ: Virtual Reality Neuroscientific Questionnaire

## Data Availability

The data sets generated during or analyzed during this study are available from the corresponding author upon request.

## References

[1] Koutsiana E, Ladakis I, Fotopoulos D, Chytas A, Kilintzis V, Chouvarda I (2020). Serious gaming technology in upper extremity rehabilitation: scoping review. JMIR Serious Games.

[2] Fidopiastis CM, Rizzo AA, Rolland JP (2010). User-centered virtual environment design for virtual rehabilitation. J Neuroeng Rehabil.

[3] Domínguez-Téllez P, Moral-Muñoz JA, Salazar A, Casado-Fernández E, Lucena-Antón D (2020). Game-based virtual reality interventions to improve upper limb motor function and quality of life after stroke: systematic review and meta-analysis. Games Health J.

[4] Vieira C, Ferreira da Silva Pais-Vieira C, Novais J, Perrotta A (2021). Serious game design and clinical improvement in physical rehabilitation: systematic review. JMIR Serious Games.

[5] Tatla SK, Shirzad N, Lohse KR, Virji-Babul N, Hoens AM, Holsti L, Li LC, Miller KJ, Lam MY, Van der Loos HM (2015). Therapists' perceptions of social media and video game technologies in upper limb rehabilitation. JMIR Serious Games.

[6] Patsaki I, Dimitriadi N, Despoti A, Tzoumi D, Leventakis N, Roussou G, Papathanasiou A, Nanas S, Karatzanos E (2022). The effectiveness of immersive virtual reality in physical recovery of stroke patients: a systematic review. Front Syst Neurosci.

[7] Levac D, Glegg S, Colquhoun H, Miller P, Noubary F (2017). Virtual reality and active videogame-based practice, learning needs, and preferences: a cross-Canada survey of physical therapists and occupational therapists. Games Health J.

[8] Rose T, Nam CS, Chen KB (2018). Immersion of virtual reality for rehabilitation - review. Appl Ergon.

[9] Pizzoli SF, Mazzocco K, Triberti S, Monzani D, Alcañiz Raya ML, Pravettoni G (2019). User-centered virtual reality for promoting relaxation: an innovative approach. Front Psychol.

[10] Reis A, Lains J, Paredes V, Filipe V, Abrantes C, Ferreira F, Mendes R, Amorim P, Barroso J (2016). Developing a system for post-stroke rehabilitation: an exergames approach. Proceedings of the 10th International Conference, UAHCI 2016, Held as Part of HCI International 2016 on Universal Access in Human-Computer Interaction, Users and Context Diversity.

[11] Leung RC, Zhang H, Tao X (2017). Serious game design for stroke rehabilitation. Int J Inf Technol.

[12] Nelson CR, Gabbard JL, Moats JB, Mehta RK (2022). User-centered design and evaluation of ARTTS: an augmented reality triage tool suite for mass casualty incidents. Proceedings of the 2022 IEEE International Symposium on Mixed and Augmented Reality.

[13] DeSmet A, Thompson D, Baranowski T, Palmeira A, Verloigne M, De Bourdeaudhuij I (2016). Is participatory design associated with the effectiveness of serious digital games for healthy lifestyle promotion? A meta-analysis. J Med Internet Res.

[14] Peláez-Vélez FJ, Eckert M, Gacto-Sánchez M, Martínez-Carrasco Á (2023). Use of virtual reality and videogames in the physiotherapy treatment of stroke patients: a pilot randomized controlled trial. Int J Environ Res Public Health.

[15] Montoya MF, Villada JF, Cardona JE, Gallo OA, López JF (2022). Diseño contextual para la creación de videojuego basado en Realidad Virtual usado en terapia de rehabilitación física en personas con accidente cerebrovascular. Rev EIA.

[16] Bui T, King C, Llado A, Lee D, Leong G, Paraparum A, Li I, Scrivener K (2019). App-based supplemental exercise during inpatient orthopaedic rehabilitation increases activity levels: a pilot randomised control trial. Pilot Feasibility Stud.

[17] Henrique PP, Colussi EL, De Marchi AC (2019). Effects of exergame on patients' balance and upper limb motor function after stroke: a randomized controlled trial. J Stroke Cerebrovasc Dis.

[18] Burke JW, McNeill MD, Charles DK, Morrow PJ, Crosbie JH, McDonough SM (2009). Optimising engagement for stroke rehabilitation using serious games. Vis Comput.

[19] Corbetta D, Imeri F, Gatti R (2015). Rehabilitation that incorporates virtual reality is more effective than standard rehabilitation for improving walking speed, balance and mobility after stroke: a systematic review. J Physiother.

[20] Charles D, Holmes D, Charles T, McDonough S, Rea PM (2020). Virtual reality design for stroke rehabilitation. Biomedical Visualisation.

[21] Duque E, Fonseca G, Vieira H, Gontijo G, Ishitani L (2019). A systematic literature review on user centered design and participatory design with older people. Proceedings of the 18th Brazilian Symposium on Human Factors in Computing Systems.

[22] Faas D, Bao Q, Frey DD, Yang MC (2014). The influence of immersion and presence in early stage engineering designing and building. Des Comput Cognit.

[23] Herrlich M, Smeddinck J, Runge N, Malaka R (2016). Applying human-centered design to develop motivating exergames. Proceedings of the 2016 Mensch und Computer Workshop.

[24] Horsham C, Dutton-Regester K, Antrobus J, Goldston A, Price H, Ford H, Hacker E (2021). A virtual reality game to change sun protection behavior and prevent cancer: user-centered design approach. JMIR Serious Games.

[25] Javaid M, Haleem A (2020). Virtual reality applications toward medical field. Clin Epidemiol Glob Health.

[26] Jerald J (2015). The VR book: Human-Centered Design for Virtual Reality.

[27] Laver KE, Lange B, George S, Deutsch JE, Saposnik G, Crotty M (2017). Virtual reality for stroke rehabilitation. Cochrane Database Syst Rev.

[28] Langener S, Klaassen R, VanDerNagel J, Heylen D (2022). Immersive virtual reality avatars for embodiment illusions in people with mild to borderline intellectual disability: user-centered development and feasibility study. JMIR Serious Games.

[29] Schell J (2008). The Art of Game Design: A Target Book.

[30] Duval J, Thakkar R, Du D, Chin K, Luo S, Elor A, El-Nasr MS, John M (2022). Designing spellcasters from clinician perspectives: a customizable gesture-based immersive virtual reality game for stroke rehabilitation. ACM Trans Access Comput.

[31] Aguilar FA, Bautista DP, Gómez A, Toledo GT, García OS (2022). User-centered virtual environment for poststroke motor rehabilitation. J Med Devices.

[32] Brox E, Konstantinidis ST, Evertsen G (2017). User-centered design of serious games for older adults following 3 years of experience with exergames for seniors: a study design. JMIR Serious Games.

[33] Soegaard M, Dam RF (2012). The Encyclopedia of Human-Computer Interaction.

[34] Chu CH, Biss RK, Cooper L, Quan AM, Matulis H (2021). Exergaming platform for older adults residing in long-term care homes: user-centered design, development, and usability study. JMIR Serious Games.

[35] Muñoz J, Mehrabi S, Li Y, Basharat A, Middleton LE, Cao S, Barnett-Cowan M, Boger J (2022). Immersive virtual reality exergames for persons living with dementia: user-centered design study as a multistakeholder team during the COVID-19 pandemic. JMIR Serious Games.

[36] Mirnig AG, Meschtscherjakov A, Wurhofer D, Meneweger T, Tscheligi T (2015). A formal analysis of the ISO 9241-210 definition of user experience. Proceedings of the 33rd Annual ACM Conference Extended Abstracts on Human Factors in Computing Systems.

[37] Lima Rebêlo F, de Souza Silva LF, Doná F, Sales Barreto A, de Souza Siqueira Quintans J (2021). Immersive virtual reality is effective in the rehabilitation of older adults with balance disorders: a randomized clinical trial. Exp Gerontol.

[38] Levin MF, Weiss PL, Keshner EA (2015). Emergence of virtual reality as a tool for upper limb rehabilitation: incorporation of motor control and motor learning principles. Phys Ther.

[39] Arya KN, Pandian S, Verma R, Garg R (2011). Movement therapy induced neural reorganization and motor recovery in stroke: a review. J Bodyw Mov Ther.

[40] Della Tommasina I, Trinidad-Morales A, Martínez-Lozano P, González-de-la-Flor Á, Del-Blanco-Muñiz JÁ (2023). Effects of a dry-land strengthening exercise program with elastic bands following the Kabat D2 diagonal flexion pattern for the prevention of shoulder injuries in swimmers. Front Physiol.

[41] Dix A (2010). Human–computer interaction: a stable discipline, a nascent science, and the growth of the long tail. Interact Comput.

[42] Gonçalves A, Muñoz J, Cameirão MS, Gouveia ÉR, Sousa H, Bermúdez I Badia S (2021). The benefits of custom exergames for fitness, balance, and health-related quality of life: a randomized controlled trial with community-dwelling older adults. Games Health J.

[43] Aguirre JJ, Gómez JA (2012). Del animal laborans al homo ludens: la configuración del juego y la fiesta como voces de resistencia al poder. PACARINA, Revista Latinoamericana.

[44] Somrak A, Pogačnik M, Guna J (2021). Suitability and comparison of questionnaires assessing virtual reality-induced symptoms and effects and user experience in virtual environments. Sensors (Basel).

[45] Lloréns R, Gil-Gómez JA, Alcañiz M, Colomer C, Noé E (2015). Improvement in balance using a virtual reality-based stepping exercise: a randomized controlled trial involving individuals with chronic stroke. Clin Rehabil.

[46] Yin CW, Sien NY, Ying LA, Chung SF, Tan May Leng D (2014). Virtual reality for upper extremity rehabilitation in early stroke: a pilot randomized controlled trial. Clin Rehabil.

[47] Kourtesis P, Linnell J, Amir R, Argelaguet F, MacPherson SE (2023). Cybersickness in virtual reality questionnaire (CSQ-VR): a validation and comparison against SSQ and VRSQ. Virtual Worlds.

[48] Kazemi R, Lee SC (2023). Human Factors/Ergonomics (HFE) evaluation in the virtual reality environment: a systematic review. Int J Hum Comput Interact.

[49] Platz T, Eickhof C, Nuyens G, Vuadens P (2005). Clinical scales for the assessment of spasticity, associated phenomena, and function: a systematic review of the literature. Disabil Rehabil.

[50] Cikajlo I, Rudolf M, Mainetti R, Borghese NA (2020). Multi-exergames to set targets and supplement the intensified conventional balance training in patients with stroke: a randomized pilot trial. Front Psychol.

[51] Holtzblatt K, Beyer H (1997). Contextual Design: Defining Customer-Centered Systems.

[52] Saltiveri TG (2007). MPIu+a. Una metodología que integra la ingeniería del software, la interacción persona-ordenador y la accesibilidad en el contexto de equipos de desarrollo multidisciplinares. Universitat de Lleida.

[53] Saeedi S, Ghazisaeedi M, Rezayi S (2021). Applying game-based approaches for physical rehabilitation of poststroke patients: a systematic review. J Healthc Eng.

[54] Hung YX, Huang PC, Chen KT, Chu WC (2016). What do stroke patients look for in game-based rehabilitation: a survey study. Medicine (Baltimore).

[55] Bedar K, Bubanovich C, Rosemore J, Radford K, Taylor KL (2023). Virtual reality intervention and its impact on upper extremity function in the stroke population: a scoping review. Games Health J.

[56] Carrión-Toro M, Santorum M, Acosta-Vargas P, Aguilar J, Pérez M (2020). iPlus a user-centered methodology for serious games design. Appl Sci.

[57] Rojo A, Castrillo A, López C, Perea L, Alnajjar F, Moreno JC, Raya R (2023). PedaleoVR: usability study of a virtual reality application for cycling exercise in patients with lower limb disorders and elderly people. PLoS One.

[58] Wu J, Zeng A, Chen Z, Wei Y, Huang K, Chen J, Ren Z (2021). Effects of virtual reality training on upper limb function and balance in stroke patients: systematic review and meta-meta-analysis. J Med Internet Res.

[59] Muñoz JE, Montoya MF, Boger J, Choukou MA, Syed-Abdul S (2022). From exergames to immersive virtual reality systems: serious games for supporting older adults. Smart Home Technologies and Services for Geriatric Rehabilitation.

[60] Hussain N (2020). Virtual reality based kinematics for assessment of post-stroke upper limb function. University of Gothenburg.

[61] Santoso HB, Schrepp M, Kartono Isal RY, Yudha Utom AY, Priyogi B (2016). Measuring user experience of the student-centered e-learning environment. J Educ Online.

[62] Proffitt R, Glegg S, Levac D, Lange B (2019). End-user involvement in rehabilitation virtual reality implementation research. J Enabling Technol.

[63] Krishnan S, Mandala MA, Wolf SL, Howard A, Kesar TM (2023). Perceptions of stroke survivors regarding factors affecting adoption of technology and exergames for rehabilitation. PM R.

[64] Montoya MF, Muñoz J, Henao O (2019). Design of an upper limbs rehabilitation videogame with sEMG and biocybernetic adaptation. Proceedings of the 5th Workshop on ICTs for improving Patients Rehabilitation Research Techniques.

[65] Fregna G, Schincaglia N, Baroni A, Straudi S, Casile A (2022). A novel immersive virtual reality environment for the motor rehabilitation of stroke patients: a feasibility study. Front Robot AI.

[66] Muñoz JE, Dautenhahn K (2021). Robo ludens: a game design taxonomy for multiplayer games using socially interactive robots. ACM Trans Hum Robot Interact.

[67] Tuena C, Pedroli E, Trimarchi PD, Gallucci A, Chiappini M, Goulene K, Gaggioli A, Riva G, Lattanzio F, Giunco F, Stramba-Badiale M (2020). Usability issues of clinical and research applications of virtual reality in older people: a systematic review. Front Hum Neurosci.

[68] Lau ST, Siah RC, Dzakirin Bin Rusli K, Loh WL, Yap JY, Ang E, Lim FP, Liaw SY (2023). Design and evaluation of using head-mounted virtual reality for learning clinical procedures: mixed methods study. JMIR Serious Games.

[69] Amorim P, Santos BS, Dias P, Silva S, Martins H (2020). Serious games for stroke telerehabilitation of upper limb - a review for future research. Int J Telerehabil.

[70] Prvu Bettger J, Liu C, Gandhi DB, Sylaja PN, Jayaram N, Pandian JD (2019). Emerging areas of stroke rehabilitation research in low- and middle-income countries: a scoping review. Stroke.

[71] Ruiz-Rodriguez A, Hermens HJ, van Asseldonk EH (2023). HEROES, design of an exergame for balance recovery of stroke patients for a home environment. Extended Abstracts of the 2023 CHI Conference on Human Factors in Computing Systems.

[72] Tao G, Garrett B, Taverner T, Cordingley E, Sun C (2021). Immersive virtual reality health games: a narrative review of game design. J Neuroeng Rehabil.

