# Supplementary material for: Design of Virtual Reality Exergames for Upper Limb Stroke Rehabilitation Following Iterative Design Methods: Usability Study
Source: JMIR Serious Games. 2024 Jan 11;12:e48900. doi: 10.2196/48900 (PMC10811592; doi:10.2196/48900)
Supplement: Multimedia Appendix 1 [file games_v12i1e48900_app1.docx]

**Multimedia Appendix 1**

Users in the Play Tests

| Play Test | Person with Stroke | Gender | Age | Impairment | Time With Stroke | Ashworth Spasticity |
| --- | --- | --- | --- | --- | --- | --- |
| First | U1 | F | 54 | Right hemiparesis | 7 meses | 1 |
|  | U2 | F | 46 | Right hemiparesis | 2 years | 2 |
| Second | U3 | M | 20 | Right hemiparesis | 20 years | 1 |
|  | U4 | F | 51 | Quadriparesis | 5 years | 1 |
|  | U5 | M | 68 | Right hemiparesis | 4 years | 2 |
| Third | U6 | F | 63 | Left-sided hemiparesis | 17 months | 1 |
|  | U7 | F | 24 | Right hemiparesis | 6 months | 1 |
| Fourth | U8 | M | 51 | Right hemiparesis | 25 years | 1 |
|  | U9 | M | 42 | Right hemiparesis | 5 years | 1 |
