# Supplementary material for: Design of Virtual Reality Exergames for Upper Limb Stroke Rehabilitation Following Iterative Design Methods: Usability Study
Source: JMIR Serious Games. 2024 Jan 11;12:e48900. doi: 10.2196/48900 (PMC10811592; doi:10.2196/48900)
Supplement: Multimedia Appendix 2 [file games_v12i1e48900_app2.docx]

**Multimedia Appendix 2**

Demographic data of the user’s study pilot

| User | Gender | Age | Motor Impairment | Years With Stroke |
| --- | --- | --- | --- | --- |
| 1 | F | 53 | Quadriparesis | 5 |
| 2 | F | 57 | Left Side Hemiparesis | 3 |
| 3 | M | 50 | Right Side Hemiparesis | 3 |
| 4 | F | 58 | Right Side Hemiparesis | 1 |
| 5 | F | 75 | Right Side Hemiparesis | 20 |
| 6 | M | 56 | Left Side Hemiparesis | 24 |
| 7 | F | 58 | Right Side Hemiparesis | 2 |
| 8 | F | 55 | Right Side Hemiparesis | 6 Month |
| 9 | M | 67 | Right Side Hemiparesis | 1 |
| 10 | M | 52 | Right Side Hemiparesis | 2 |
