# Supplementary material for: Design of Virtual Reality Exergames for Upper Limb Stroke Rehabilitation Following Iterative Design Methods: Usability Study
Source: JMIR Serious Games. 2024 Jan 11;12:e48900. doi: 10.2196/48900 (PMC10811592; doi:10.2196/48900)
Supplement: Multimedia Appendix 3 [file games_v12i1e48900_app3.docx]

**Multimedia Appendix 3**

Changes made throughout the iteration process of the playtests

| Scenario | Prototype | Latest Prototype |
| --- | --- | --- |
| The Carpenter | The main scene for box hammering | The main scene for box hammering |
|  | Widely scattered objects in the virtual scene | Move Calibration Scene |
|  | No specific content | Difficulty Calibration Scene |
|  | Interaction without break times interaction with both arms | Option to choose which arm to start playing with |
|  |  | Objects are ideal positions in the virtual scene |
|  |  | With content specific to real-life |
|  |  | Interaction with break times |
|  |  | Interaction with both arms |
|  |  | Concatenation with the other scenarios |
| Tejo | The main scene to throw the shuffleboard | The main scene to throw the shuffleboard |
|  | No specific content | Move Calibration Scene |
|  | Interaction without break times | Difficulty Calibration Scene |
|  | Interaction with both arms | Option to choose which arm to start playing with |
|  |  | With sport-specific content |
|  |  | Interaction with break times |
|  |  | Interaction with both arms |
|  |  | Concatenation with the other scenarios |
| The farmer | The main scene to cutting bushes horse down avatar | With movement calibration scene |
|  | Widely scattered objects in the virtual scene | With the option to choose which arm to start playing with |
|  | The appearance of objects at the wrong time | The main scene is cutting bushes |
|  | No break times | Avatar on top of the horse |
|  | Interaction with both arms | Objects are in ideal positions in the virtual scene |
|  |  | Interaction with break times |
|  |  | Interaction with both arms |
|  |  | Concatenation with the other scenarios |
