# Supplementary material for: Design of Virtual Reality Exergames for Upper Limb Stroke Rehabilitation Following Iterative Design Methods: Usability Study
Source: JMIR Serious Games. 2024 Jan 11;12:e48900. doi: 10.2196/48900 (PMC10811592; doi:10.2196/48900)

**Multimedia Appendix 4**

Scenario sketches

A storyboard consists of a series of drawings or images arranged in sequential illustrations, depicting how a certain system will be used to perform a certain task [38]. We proposed the main scenes of the exergames, the functionality of the buttons, and the general story of the exergames through a series of sketches, as can be seen in the figure. As shown in these sketches, we envisioned the exergames with three main scenes where the players, who have suffered a stroke, must perform three different activities: hammering, playing a throwing game in a traditional game called “tejo”, and cutting bushes while riding a horse in a sugar cane field.

Scenario sketches of the exergames, and main scenes. A) hammering, B) “Tejo”, C) bush felling on horseback


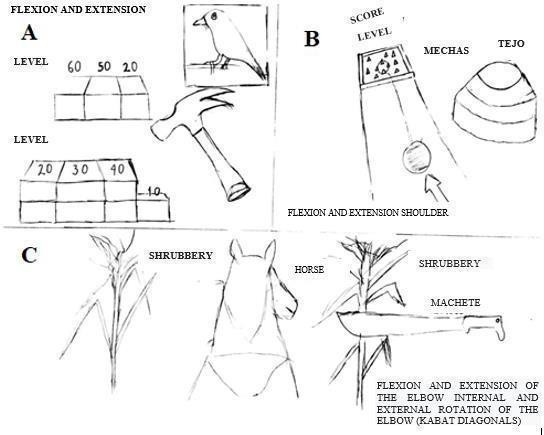

Supplement: Multimedia Appendix 4 [file games_v12i1e48900_app4.docx]
